# Supplementary material for: T-cell protrusions enable fast, localised initiation of chimeric antigen receptor signalling
Source: EMBO J. 2026 Apr 21;45(10):3337–63. doi: 10.1038/s44318-026-00773-5 (PMC13187322; doi:10.1038/s44318-026-00773-5)
Supplement: Supplementary file 1 — Table EV1 [file 44318_2026_773_MOESM1_ESM.docx]

| **Primer Name** | **Primer Sequence (5’-3’)** |
| --- | --- |
| **Amplification of SNAP tag from pSNAPf vector** | |
| NheI-SNAP-sense | atcgctagcatggacaaagactgcgaaatgaagc |
| EcoRI-SNAP-G/Slinker-antisense 5 | cagctgaattccggccgctgcctccgctgccgcttccctcgagggatcctggcgcgcct |
| **Amplification of Halo fragment from (L-selectin-LAP-Halo-polyAG418)** | |
| BamHI-Halo se | TGTTCCGGATCCATGGCAGAAATCGGTACTGGCTTTC |
| Halo-NotI ase | GGAACAGCGGCCGCTCAGCCGGAAATCTCGAGCGTCGACAGC |
| **Amplification of NheI-Halo-2xHA-EcoRI fragment for LAT HR plasmid from pHalo-N1ARF1-Halo** | |
| EcoRI-PolyA sense | ctgatGCTAGCATGGCAGAAATCGGTACTGGCTTTC |
| Halo-2HA-EcoRI antisense | GCTGGCTGTCGACGCTCGAGATTTCCGGCTACCCATACGATGTTCCAGATTACGCTTACCCATACGATGTTCCAGATTACGCTTGAGAATTCtcgta |
| **Amplification of EcorRI-polyA-G418-EcoRI from pHalo-N1ARF1-Halo fragment for the LAT HR plasmid** | |
| EcoRI-PolyA sense | atgtcGAATTCccgcgactctagatcataatcagc |
| EcoRI-G418 antisense | tcatgGAATTCtttattctgtctttttattgccgtc |
| **Amplification of EcoRI-polyA-HindIII fragment from pHalo-N1ARF1-Halo for ZAP-70 plasmid** | |
| EcoRI-polyA se | AATTGAATTCCCGCGACTCTAGATCATAATCAGCCATACC |
| polyA-HindIII ase | tctctcaagcttcttacaatttacgccttaagatacA |
| **Amplification of HindIII-G418-BamHI fragment from pHalo-N1ARF1-Halo for ZAP-70 plasmid** | |
| HindIII-spacer-G418 se | gagagaaagcttcctgaggcggaaagaaccagctgtggaatgtgtgtcagttag |
| G418 BamHI anti | AATTGGATCCTTATTCTGTCTTTTTATTGCCGTCATAGC |

**Table EV1:** Primers used for cloning
