# Supplementary material for: T-cell protrusions enable fast, localised initiation of chimeric antigen receptor signalling
Source: EMBO J. 2026 Apr 21;45(10):3337–63. doi: 10.1038/s44318-026-00773-5 (PMC13187322; doi:10.1038/s44318-026-00773-5)
Supplement: Supplementary file 2 — Table EV2 [file 44318_2026_773_MOESM2_ESM.docx]

| **gBlock Name** | **gBlock Sequence (5’-3’)** | **Legend** |
| --- | --- | --- |
| HER2-CAR for insertion into pEGFP-N1 | TACCGGACTCAGATCTCGAGCTCAAGCTTCGAATTC**ATGGACTGGATCTGGCGCATCCTGTTTCTCGTGGGAGCCGCCACAGGCGCCCATGAGGTACAACTGCAGCAGTCTGGACCTGAACTGAAGAAGCCTGGAGAGACAGTCAAGATCTCCTGCAAGGCCTCTGGGTATCCTTTCACAAACTATGGAATGAACTGGGTGAAGCAGGCTCCAGGACAGGGTTTAAAGTGGATGGGCTGGATTAACACCTCCACTGGAGAGTCAACATTTGCTGATGACTTCAAGGGACGGTTTGACTTCTCTTTGGAAACCTCTGCCAACACTGCCTATTTGCAGATCAACAACCTCAAAAGTGAAGACATGGCTACATATTTCTGTGCAAGATGGGAGGTTTACCACGGCTACGTTCCTTACTGGGGCCAAGGGACCACGGTCACCGTTTCCTCTGGAGGcGGAGGcTCTGGTGGCGGTGGCTCCGGCGGTGGCGGTTCTGACATCCAGCTGACCCAGTCTCACAAATTCCTGTCCACTTCAGTAGGAGACAGGGTCAGCATCACCTGCAAGGCCAGTCAGGATGTGTATAATGCTGTTGCCTGGTATCAACAGAAACCAGGACAATCTCCTAAACTTCTGATTTACTCGGCATCCTCCCGGTACACTGGAGTCCCTTCTCGCTTCACTGGCAGTGGCTCTGGGCCGGATTTCACTTTCACCATCAGCAGTGTGCAGGCTGAAGACCTGGCAGTTTATTTCTGTCAGCAACATTTTCGTACTCCATTCACGTTCGGCTCGGGGACAAAATTGGAGATCAAAGCTCTAGATCTCGAGCCCAAATCTTGTGACAAAACTCACACATGCCCACCGTGCCCGGACCCCAAATTTTGGGTGCTGGTGGTGGTTGGTGGAGTCCTGGCTTGCTATAGCTTGCTAGTAACAGTGGCCTTTATTATTTTCTGGGTGAGGAGTAAGAGGAGCAGGCTCCTGCACAGTGACTACATGAACATGACTCCACGTCGACCAGGTCCAACTCGCAAGCATTACCAGCCCTATGCCCCACCACGCGACTTCGCAGCCTATCGCTCCAGAGTGAAGTTCAGCAGGAGCGCAGACGCCCCCGCGTACCAGCAGGGCCAGAACCAGCTCTATAACGAGCTCAATCTAGGACGAAGAGAGGAGTACGATGTTTTGGACAAGAGACGTGGCCGGGACCCTGAGATGGGGGGAAAGCCGAGAAGGAAGAACCCTCAGGAAGGCCTGTACAATGAACTGCAGAAAGATAAGATGGCGGAGGCCTACAGTGAGATTGGGATGAAAGGCGAGCGCCGGAGGGGCAAGGGGCACGATGGCCTTTACCAGGGTCTCAGTACAGCCACCAAGGACACCTACGACGCCCTTCACATGCAGGCCCTGCCCCCTCGC**GGATCAGGGTCAGGCTCTGGTTCCGGCTCACGGGATCCACCGGTCGCCACCATGGTGAGCAAGGGCG | 30 bp overlap with plasmid of insertion;  EcoRI (first), BamHI (second);  **leader peptide**; **scFvFRP5;**  **A silent mutations to prevent the repeat of the sequence “ggcggtggcggttct”**;  **short hinge**;  **linker**;  **C silent mutation to eliminate unwanted BamHI site**;  **CD28 transmembrane and signaling domain,**  **A silent mutations to reduce number of G/C bases by manufacturer request;**  **AGAG zeta signaling domain;**  GGA linker; |
| Truncated HER2-CAR for insertion into pEGFP-N1 | TACCGGACTCAGATCTCGAGCTCAAGCTTCGAATTC**ATGGACTGGATCTGGCGCATCCTGTTTCTCGTGGGAGCCGCCACAGGCGCCCATGAGGTACAACTGCAGCAGTCTGGACCTGAACTGAAGAAGCCTGGAGAGACAGTCAAGATCTCCTGCAAGGCCTCTGGGTATCCTTTCACAAACTATGGAATGAACTGGGTGAAGCAGGCTCCAGGACAGGGTTTAAAGTGGATGGGCTGGATTAACACCTCCACTGGAGAGTCAACATTTGCTGATGACTTCAAGGGACGGTTTGACTTCTCTTTGGAAACCTCTGCCAACACTGCCTATTTGCAGATCAACAACCTCAAAAGTGAAGACATGGCTACATATTTCTGTGCAAGATGGGAGGTTTACCACGGCTACGTTCCTTACTGGGGCCAAGGGACCACGGTCACCGTTTCCTCTGGAGGcGGAGGcTCTGGTGGCGGTGGCTCCGGCGGTGGCGGTTCTGACATCCAGCTGACCCAGTCTCACAAATTCCTGTCCACTTCAGTAGGAGACAGGGTCAGCATCACCTGCAAGGCCAGTCAGGATGTGTATAATGCTGTTGCCTGGTATCAACAGAAACCAGGACAATCTCCTAAACTTCTGATTTACTCGGCATCCTCCCGGTACACTGGAGTCCCTTCTCGCTTCACTGGCAGTGGCTCTGGGCCGGATTTCACTTTCACCATCAGCAGTGTGCAGGCTGAAGACCTGGCAGTTTATTTCTGTCAGCAACATTTTCGTACTCCATTCACGTTCGGCTCGGGGACAAAATTGGAGATCAAAGCTCTAGATCTCGAGCCCAAATCTTGTGACAAAACTCACACATGCCCACCGTGCCCGGACCCCAAA**TTTTGGGTGCTGGTGGTGGTTGGTGGAGTCCTGGCTTGCTATAGCTTGCTAGTAACAGTGGCCTTTATTATTTTCTGGGTGGGATCCACCGGTCGCCACCATGGTGAGCAAGGGCG | overlap with pGFPN1;  EcoRI (first) , BamHI (second); **leader peptide;**  **GAGG scFvFRP5**  **A silent mutations to prevent the repeat of a sequence;**  **GATC short hinge**;  **GATC linker amino acids**,  **C silent mutation to get rid of a BamHI site**;  TTTTG GS linker |
| LifeAct for insertion into pEGFP-N1 | TACCGGACTCAGATCTCGAGCTCAAGCTTCGAATTCGCCACGATGGGTGTTGCGGACCTTATCAAGAAATTTGAGAGCATAAGCAAGGAGGAGGGTGATCCTCCTGTTGCTACTGCGGATCCACCGGTCGCCACCATGGTGAGCAAGGGCGA | Overlap 30bp with eGFPN1 vector;  EcoRI (first), BamHI (second);  Kozak sequence;  Lifeact;  GDPP linker;  GC to keep in frame |

**Table EV2:** gBlocks used for cloning
