# Supplementary material for: T-cell protrusions enable fast, localised initiation of chimeric antigen receptor signalling
Source: EMBO J. 2026 Apr 21;45(10):3337–63. doi: 10.1038/s44318-026-00773-5 (PMC13187322; doi:10.1038/s44318-026-00773-5)
Supplement: Supplementary file 3 — Table EV3 [file 44318_2026_773_MOESM3_ESM.docx]

| Targeted locus | Guide RNA sequence  (5’-3’, PAM site underlined) | Vector |
| --- | --- | --- |
| Lck (+ strand)  (gene ID 3932) | CAGCCTTGAGAGGCCTTGAGAGG | pX330 |
| ZAP-70 (+ strand)  (gene ID 7535) | CCCAGGGGAGCCCTCCACGCCGG | pX330 |
| LAT (+ strand)  (gene ID 27040) | GACAGGGCAGGCCTCTCACCGGGAGG | pX459 |
| PTPRC/CD45 (- strand)  (gene ID 5788) | CATAGGAAAAGACATAAATGAGG | pX330 |

**Table EV3:** gRNA sequences used for gene-editing
