# Supplementary material for: T-cell protrusions enable fast, localised initiation of chimeric antigen receptor signalling
Source: EMBO J. 2026 Apr 21;45(10):3337–63. doi: 10.1038/s44318-026-00773-5 (PMC13187322; doi:10.1038/s44318-026-00773-5)
Supplement: Supplementary file 4 — Appendix [file 44318_2026_773_MOESM4_ESM.pdf]

Appendix for "T-cell protrusions enable fast, localised initiation of chimeric antigen receptor signalling"

Table of contents

Appendix Figure S1 2

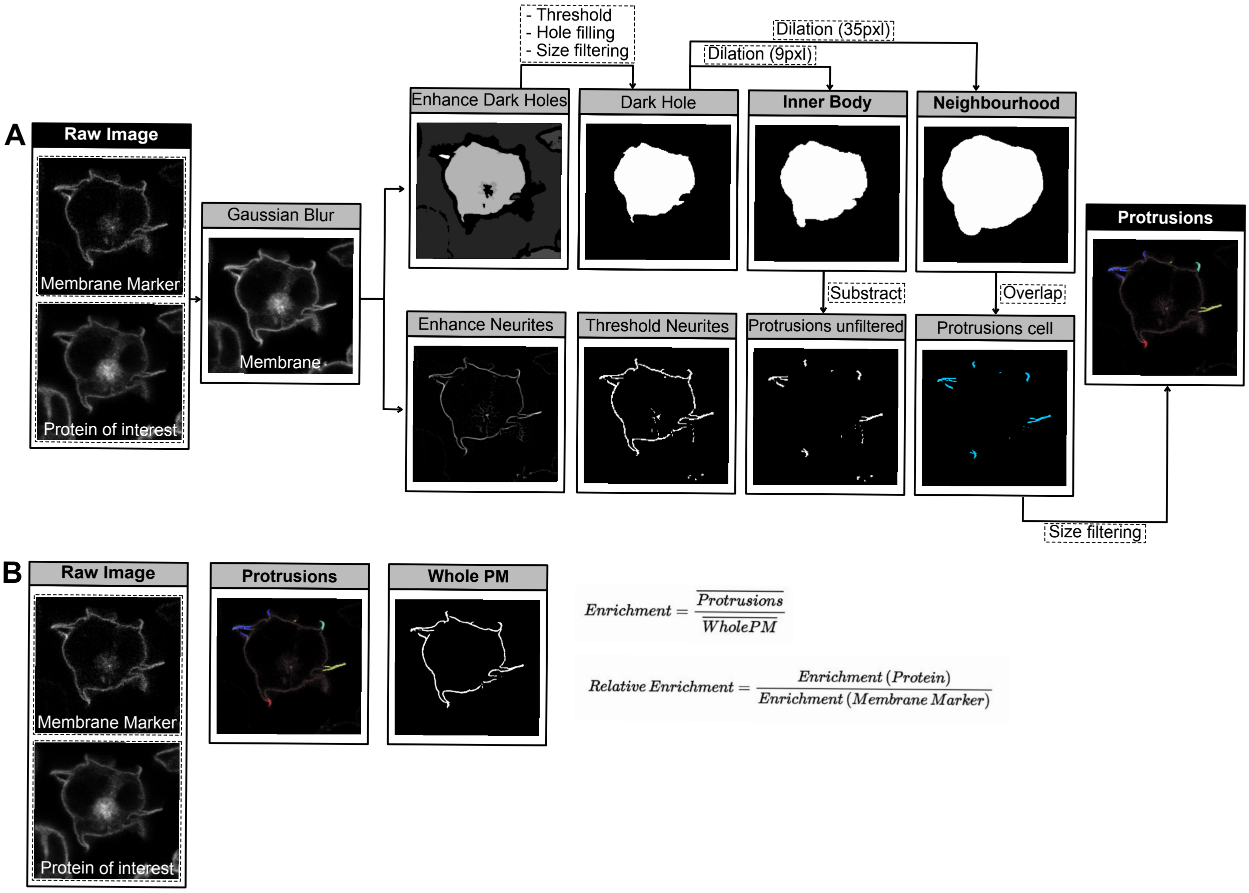

Appendix Figure S1: Segmentation of protrusive structures.

A) Description of the protrusion segmentation pipeline. Two-colour images of proteins localising to the plasma membrane of Jurkat T cells were used to segment protrusions using a custom CellProfiler 4 pipeline. First, the two membrane channels were averaged and Gaussian-blurred to generate a "membrane" image. (Left row) For inner body detection, dark holes were enhanced, thresholded, size-filtered and filled. The inner body was then dilated 9 pixels, so it covered the main body membrane, and 35 pixels to obtain the cell neighbourhood. (Right row) For membrane detection, neurite-like structures were enhanced and thresholded to obtain an unfiltered picture of the membrane. Unfiltered protrusions were obtained by subtracting the dilated inner body from the thresholded neurites. Protrusions outside the neighbourhood were filtered out, and size filtering was then applied for discarding single-pixel objects.
B) Calculation of *enrichment* and *relative enrichment* of a protein of interest in a membrane set
