# Supplementary material for: T-cell protrusions enable fast, localised initiation of chimeric antigen receptor signalling
Source: EMBO J. 2026 Apr 21;45(10):3337–63. doi: 10.1038/s44318-026-00773-5 (PMC13187322; doi:10.1038/s44318-026-00773-5)
Supplement: Supplementary file 15 — Expanded View Figures [file 44318_2026_773_MOESM15_ESM.pdf]

Expanded View Figures

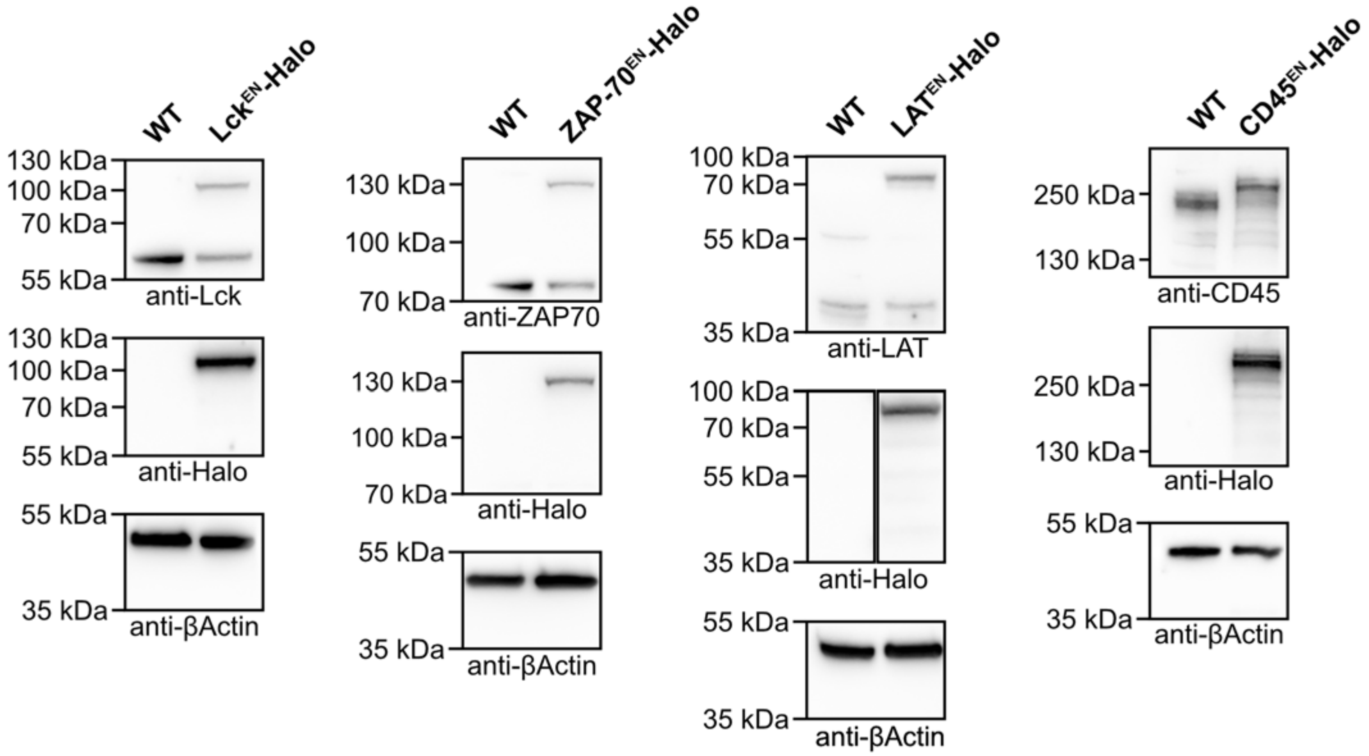

Figure EV1. Validation of knock-in (KI) cell lines via western blot.

Western blots of lysates of Jurkat T cell lines expressing endogenously Halo-tagged Lck, ZAP-70, LAT or CD45. Primary antibodies used for each immunoblot are shown below each crop. Full blots are provided in the source data file. All fusion proteins display the correct shift in molecular weight, corresponding to the molecular weight of the linker (short GS linker for Lck and LAT; long 70aa LAP linker for CD45 and ZAP-70), Halo and an epitope tag (2xalfa or 2xHA). For the CD45 KI, all alleles appear to be edited as shown by a full shift of the band corresponding to WT CD45. For the Lck, ZAP-70 and LAT KI cell lines, only ~50% of alleles are edited.

© The Author(s)

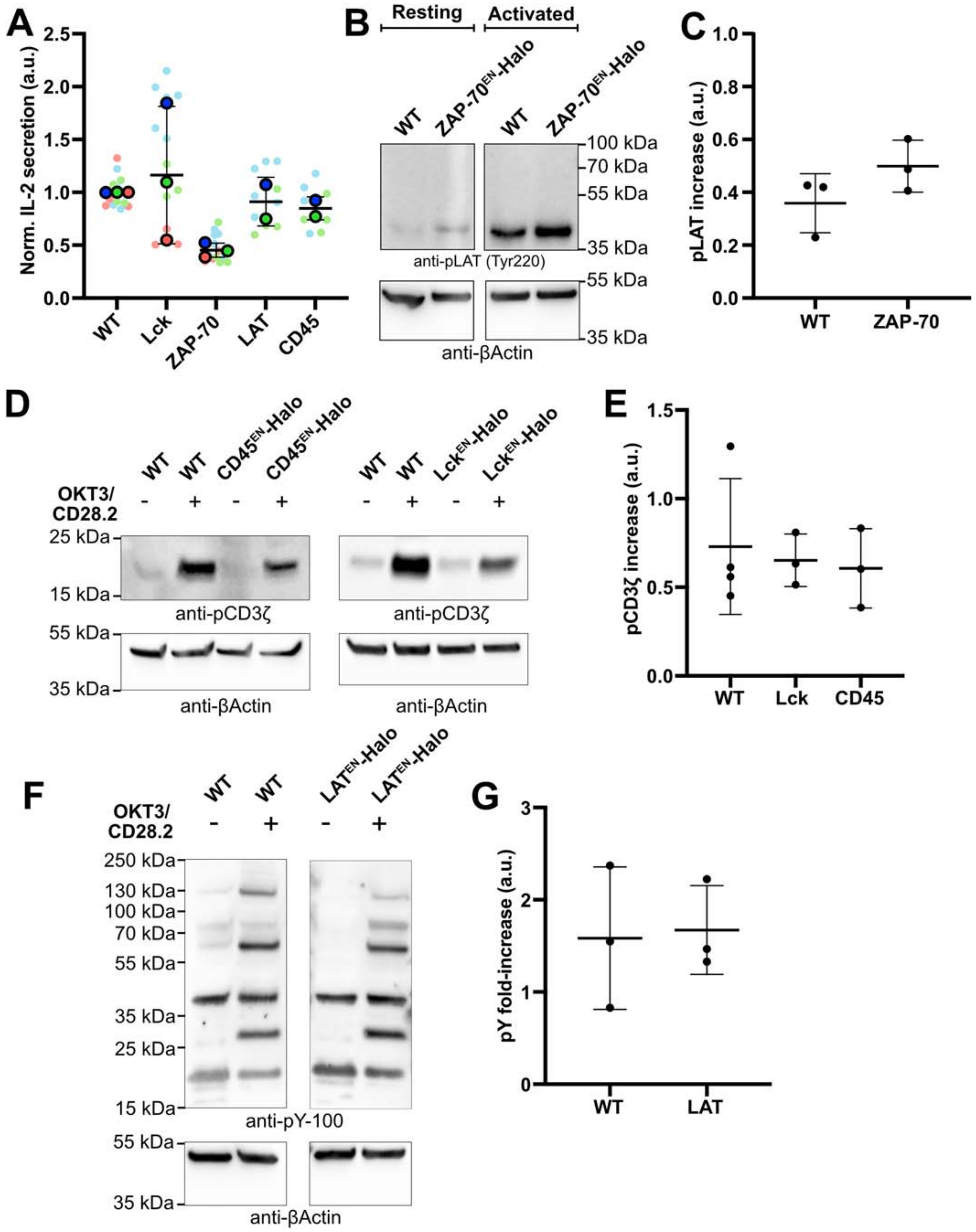
A
Norm. IL-2 secretion (a.u.)
2.5
2.0
1.5
1.0
0.5
0.0
WT
Lck
ZAP-70
LAT
CD45
B
Resting
Activated
WT
ZAP-70EN-Halo
WT
ZAP-70EN-Halo
100 kDa
70 kDa
55 kDa
35 kDa
anti-pLAT (Tyr220)
55 kDa
35 kDa
anti-βActin
C
pLAT increase (a.u.)
1.0
0.8
0.6
0.4
0.2
0.0
WT
ZAP-70
D
WT
WT
CD45EN-Halo
CD45EN-Halo
WT
WT
LckEN-Halo
LckEN-Halo
OKT3/
CD28.2
- + - +
- + - +
25 kDa
15 kDa
anti-pCD3ζ
anti-pCD3ζ
55 kDa
35 kDa
anti-βActin
anti-βActin
E
pCD3ζ increase (a.u.)
1.5
1.0
0.5
0.0
WT
Lck
CD45
F
WT
WT
LATEN-Halo
LATEN-Halo
OKT3/
CD28.2
- + - +
250 kDa
130 kDa
100 kDa
70 kDa
55 kDa
35 kDa
25 kDa
15 kDa
anti-pY-100
55 kDa
35 kDa
anti-βActin
G
pY fold-increase (a.u.)
3
2
1
0
WT
LAT

© The Author(s)

Figure EV2. Validation of KI cell lines functionality.

(**A**) IL-2 secretion was assessed in supernatants of WT and KI Jurkat T cells cultured in dishes coated with OKT3 and CD28.2 antibodies for 24 h. IL-2 secretion is normalised to the mean secretion from unmodified Jurkat T cells from the same day. Replicates are shown in different colours, and each small dot represents the normalised concentration of IL-2 collected from the supernatant of one well. $n = 12$ 8-well supernatant (for WT), $n = 14$ well supernatants (for Lck), $n = 13$ well supernatants (for ZAP-70), $n = 10$ well supernatants (for LAT) and $n = 10$ well supernatants (for CD45) from three (WT, Lck, ZAP-70) or two (LAT, CD45) independent experiments were analysed. The graph shows mean values and s.d. error bars. P values from paired t tests are 0.7050 (WT/Lck), 0.050 (WT/ZAP-70), 0.6840 (WT/LAT) and 0.2970 (WT/CD45). (**B**) Cell lysates derived from the ZAP-70^{EN}-Halo cell line or the wildtype Jurkats either resting or activated in OKT3/CD28.2 coated dishes were immunoblotted using anti-pLAT and anti-β-actin antibodies. (**C**) Increase of pLAT signal normalised to β-actin signal upon activation. $n = 3$ experiments are plotted. The graph shows mean values and s.d. error bars. P value from paired t test is 0.2219. (**D**) Cell lysates derived from the WT, Lck^{EN}-Halo and CD45^{EN}-Halo, either resting or activated in OKT3/CD28.2 coated dishes, were immunoblotted using anti-pCD3ζ and anti-β-actin antibodies. (**E**) Increase pCD3ζ signal normalised to β-actin signal upon activation. $n = 3$ experiments are plotted. The graph shows mean values and s.d. error bars. P values from paired t tests are (WT/Lck) 0.7159 and (WT/CD45) 0.5158. (**F**) Cell lysates derived from the WT and LAT^{EN}-Halo, either resting or activated in OKT3/CD28.2-coated dishes, were immunoblotted using anti-phosphotyrosine (pY) and anti-β-actin antibodies. (**G**) Increase pY signal normalised to β-actin signal upon activation. $n = 3$ experiments are plotted. The graph shows mean values and s.d. error bars. P value from paired t test is 0.8747.

© The Author(s)

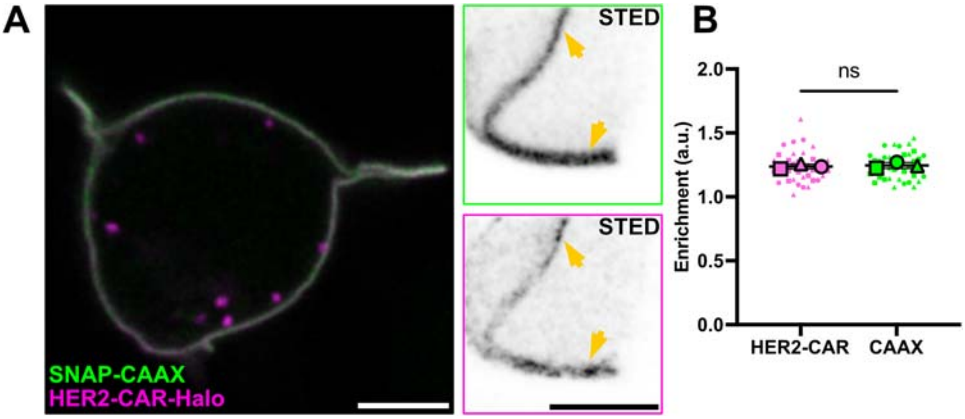

Figure EV3. HER2-CAR shows no preferential localisation to actin protrusions in resting conditions.

(**A**) Live-cell confocal (magenta and green) and STED images (inverted greyscale) of a Jurkat T cell expressing HER2-CAR-Halo labelled with CA-JFX_{650} and SNAP-CAAX labelled with BG-JF_{571}. Arrows highlight small HER2-CAR clusters localised either to the main body membrane or to an actin protrusion. (**B**) *Enrichment* of HER2-CAR tagged with Halo in protrusions. In total, $n = 36$ cells from three independent experiments were analysed. Replicates are shown as different shapes, and each small dot represents a single cell. The graph shows mean values and s.d. error bars. P value of paired t tests is 0.6116. CA chloroalkane (HaloTag substrate), BG benzylguanine (SNAP-tag substrate). Scale bars, 5 µm (confocal overview), 2 µm (STED images).

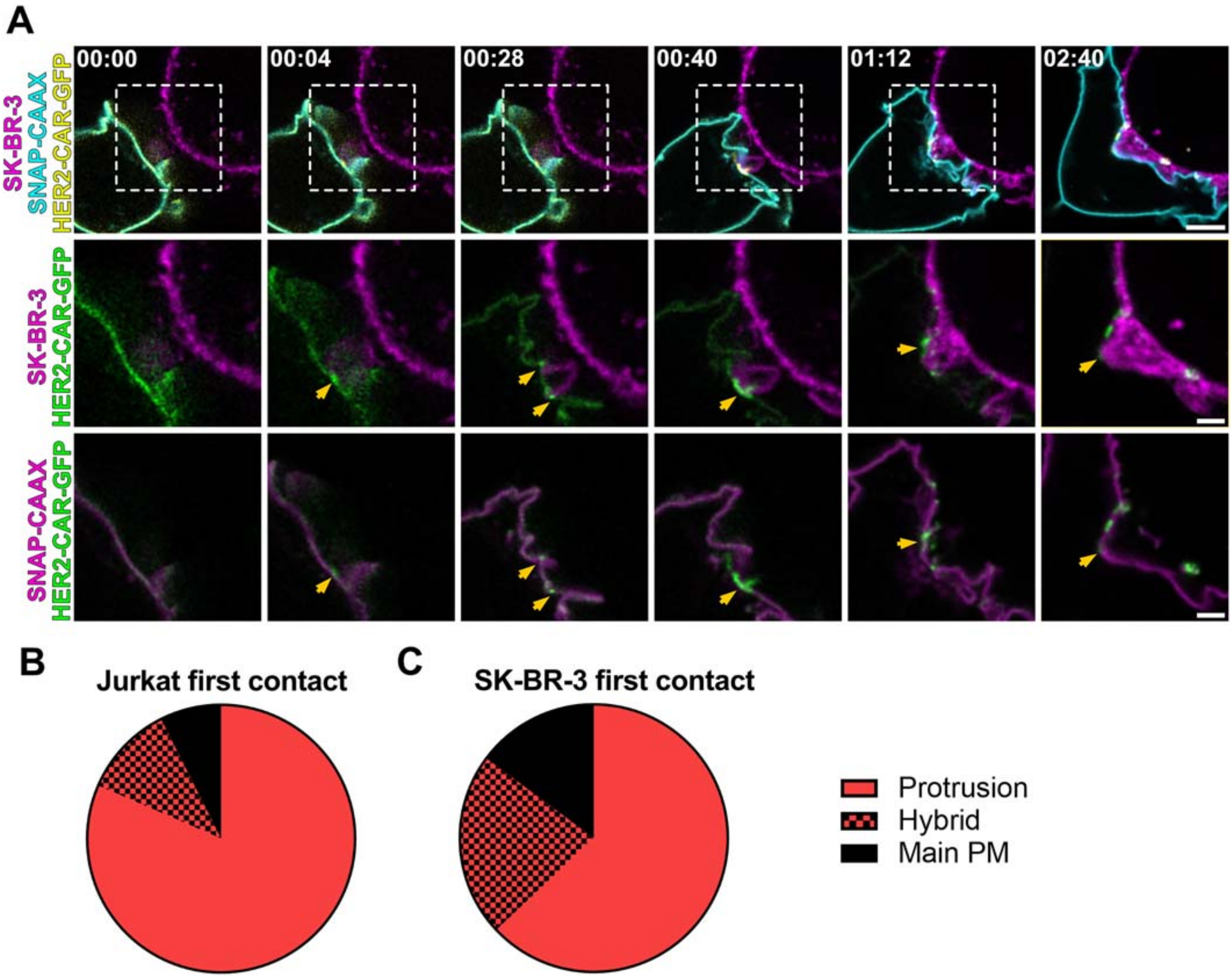

Figure EV4. Protrusions from the target cell make contact with Jurkat T cells and lead to successful CAR engagement and clustering.

(**A**) Time-lapse confocal imaging of a Jurkat T cell expressing HER2-CAR-GFP, SNAP-CAAX (labelled with BG-JF_{571}) interacting with a SK-BR-3 cell (labelled with CellMaskOrange). The dashed line describes the outline of the SK-BR-3 cell. Arrows highlight the cell–cell contact mediated by a protrusion emanating from the SK-BR-3. The contact leads to successful clustering of the HER2-CAR on the Jurkat T cell membrane. (**B**) Number of Jurkat T cell/SK-BR-3 first detected contacts mediated through Jurkat T cell protrusions, main body membrane or both at the same time. $n = 29$ from three independent experiments are represented. (**C**) Number of Jurkat T cell/SK-BR-3 first detected contacts mediated through SK-BR-3 protrusions, main body membrane or both at the same time. $n = 29$ independent cell–cell interaction events were analysed. BG benzylguanine (SNAP-tag substrate). Scale bars, 5 µm (confocal overviews), 2 µm (crops).

 © The Author(s)

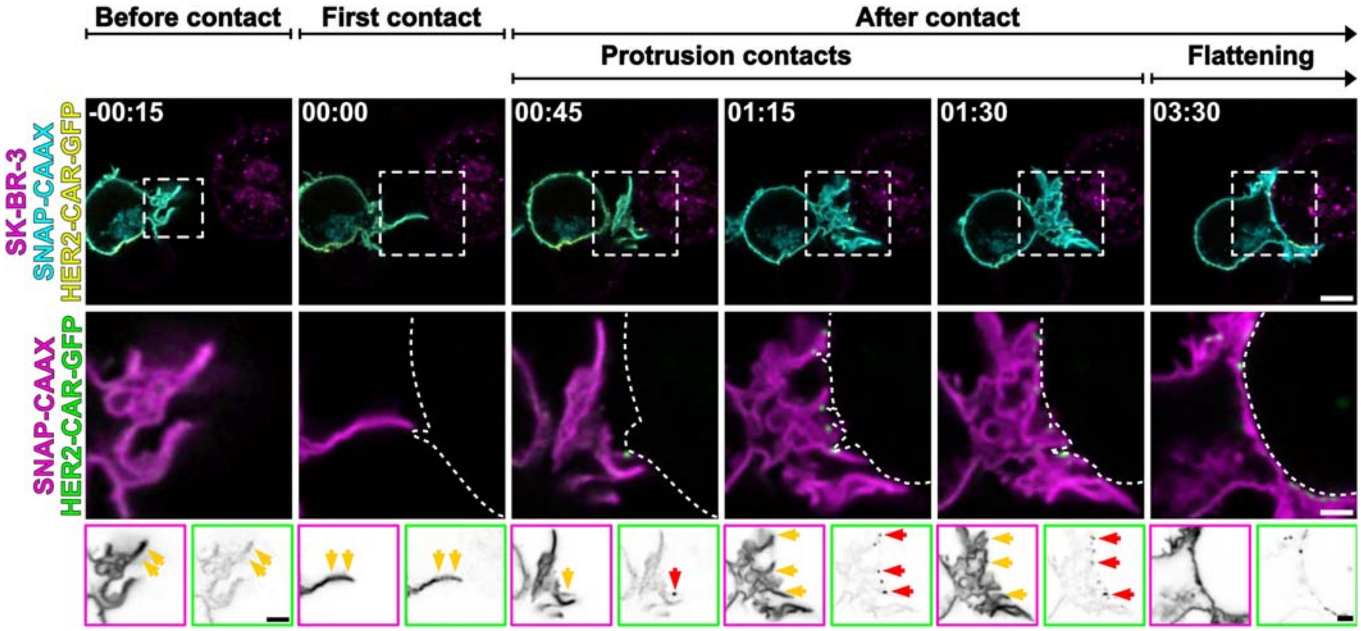

Figure EV5. Early contact between a CD4+ primary T cells and the target cell is mediated by protrusions that trigger CAR clustering and activation.

Time-lapse confocal imaging of a CD4+ human T-cell expressing HER2-CAR-GFP, SNAP-CAAX (labelled with BG-JF_{571}) interacting with a SK-BR-3 cell (labelled with CellMaskOrange). The dashed line describes the outline of the SK-BR-3 cell. HER2-CAR shows no preferential localisation to protrusions prior to first detected contact with the target at $t = 0$ s, but is enriched in protrusion contacts. BG benzylguanine (SNAP-tag substrate). Scale bars, 5 μm (confocal overviews), 2 μm (crops).

© The Author(s)
